# Multi-Cue-Based Circle Detection and Its Application to Robust Extrinsic Calibration of RGB-D Cameras

**DOI:** 10.3390/s19071539

**Published:** 2019-03-29

**Authors:** Young Chan Kwon, Jae Won Jang, Youngbae Hwang, Ouk Choi

**Affiliations:** 1 Department of Electronics Engineering, Incheon National University, Incheon 22012, Korea; yckwon@inu.ac.kr (Y.C.K.); jjwopal@inu.ac.kr (J.W.J.); 2 Intelligent Image Processing Research Center, Korea Electronics Technology Institute, Gyeonggi-do 13488, Korea; ybhwang@keti.re.kr

**Keywords:** RGB-D camera, extrinsic calibration, spherical object, circle detection, multi-cue, robust estimation

## Abstract

RGB-Depth (RGB-D) cameras are widely used in computer vision and robotics applications such as 3D modeling and human–computer interaction. To capture 3D information of an object from different viewpoints simultaneously, we need to use multiple RGB-D cameras. To minimize costs, the cameras are often sparsely distributed without shared scene features. Due to the advantage of being visible from different viewpoints, spherical objects have been used for extrinsic calibration of widely-separated cameras. Assuming that the projected shape of the spherical object is circular, this paper presents a multi-cue-based method for detecting circular regions in a single color image. Experimental comparisons with existing methods show that our proposed method accurately detects spherical objects with cluttered backgrounds under different illumination conditions. The circle detection method is then applied to extrinsic calibration of multiple RGB-D cameras, for which we propose to use robust cost functions to reduce errors due to misdetected sphere centers. Through experiments, we show that the proposed method provides accurate calibration results in the presence of outliers and performs better than a least-squares-based method.

## 1. Introduction

An RGB-D camera is a tightly-coupled pair of one depth camera and one color camera. Because of the benefits of providing color and depth information in real time, RGB-D cameras have been widely used in many computer vision and robotics tasks such as human or hand pose estimation [1,2], dense 3D modeling of the surrounding environment [3], and simultaneous localization and mapping [4].

A single RGB-D camera can capture full 3D information of a static object or environment. We can move the camera to capture multiple color and depth image pairs from different viewpoints. The pieces of 3D information of the individual depth images are then fused together by using the iterative closest point algorithm [3] or by matching features across images [4] to produce a dense 3D model of the object or the environment.

Once the RGB-D camera is fully calibrated, the acquired 3D points can be mapped to their corresponding pixels in the color images to enable texture mapping of the reconstructed 3D model. Commercially-available RGB-D cameras [5,6,7] are typically calibrated at the factory, and this paper assumes that the individual RGB-D cameras have been fully calibrated.

On the other hand, we need to use multiple RGB-D cameras to capture the 3D information of an object from different viewpoints simultaneously. To fuse the 3D points acquired by different RGB-D cameras in the reference coordinate system, extrinsic parameters between the RGB-D cameras are necessary. The extrinsic parameters, however, need to be estimated by the user, and the estimation is not an easy task without carefully-designed calibration objects and algorithms. In this paper, we propose a fully-automated method for estimating extrinsic parameters between different RGB-D cameras.

Zhang’s calibration method [8] using a planar checkerboard is the most popular choice for many users and researchers because of its implementations with automated feature extraction [9]. To use the method for extrinsic calibration, adjacent cameras need to be close enough to see the same scene features simultaneously.

From an economic standpoint, it is better to use as few RGB-D cameras as possible. With a limited number of cameras, it is effective to place the cameras sparsely so that they will see different sides of the object. In this case, the planar checkerboard is not an appropriate calibration target because it is difficult for a pair of widely-separated cameras to see the same side of the board simultaneously.

A self-calibration approach [10] uses a laser pointer to establish 2D point correspondences across widely-separated views. RGB-D cameras, however, cannot accurately measure the depth of a small object or the depth of object edges [11]. Therefore, when applying the approach to extrinsic calibration of RGB-D cameras, we should provide additional 3D information such as the distance between two cameras or the size of an object.

A spherical object [12,13,14,15,16,17,18,19,20,21] overcomes the limitations of the planar board and the laser pointer. Because the surface is smooth, the measured depth is accurate except for the edges. In addition, the spherical object is visible from widely-separated viewpoints simultaneously. The cameras generally do not see the same side of the spherical object, but the sphere centers estimated from the surface depth measurements act as 3D point correspondences across the cameras.

In previous approaches, spherical objects were usually lighted [12,14] or painted in a unique color [13,15,16,17,18,19,20]. The environment is typically assumed to be under uniform lighting conditions and is assumed to be of a different color than the spherical object. In this case, we can apply a simple threshold [12,14] or background subtraction algorithm [15,16,17,18,20] to reduce the search regions for the spherical object. We can also use the object’s color distribution to detect its projected regions in color images [15,20,21].

Assuming a studio or laboratory environment, it is possible to control lighting or the environment. However, the background subtraction algorithm may fail to reduce the search region if the environment contains objects of similar color to the sphere or is under nonuniform lighting conditions. If the sphere’s color is not distinct from the background, the color distribution-based methods [15,20,21] may also fail.

Assuming that the projected shape of the spherical object is circular, we propose a circle detection method based on region and edge cues. The projected sphere shape is generally elliptical [22]. If the camera has negligible radial distortion, our assumption roughly holds. Fortunately, color cameras equipped in RGB-D cameras generally have small radial distortion. In addition, some RGB-D cameras [6] provide aligned and undistorted pairs of color and depth images.

Our method uses only a single color image and does not rely on background subtraction. In addition, it does not rely on color distribution models [15,20]. Kwon et al. [21] used the input image to model the background color distribution so that the decision boundary between the object’s color and other colors will be automatically determined. The method is, however, vulnerable to changes in lighting, so that other objects are detected as spheres regardless of shape. Instead of the color distribution model, our method relies on color-based hierarchical segmentation that divides a given set of pixels into two disjoint subsets. One is similarly colored to the mean color of the spherical object, and the other is distant from the mean color. Circles are then detected on both subsets based on a region-and-edge-cue-based cost. To find more circles in smaller regions, the original set is recursively replaced with the similarly-colored set. Following the convention in the literature [15,16,17,18,19,20], we assume that a single sphere exists in the image, and we use the multi-cue based cost to choose the best circle. Since the proposed method essentially finds multiple circles, it is simple to extend to the detection of multiple spheres.

The detected circles may not always be accurate, and the fitted sphere centers may have large errors. To cope with false detection and errors, we use robust cost functions at every stage of the proposed extrinsic calibration procedure. By using the robust cost functions in the M-estimator-based random sampling framework [23], we do not have to find the outliers, but we can get accurate results. We demonstrate the effectiveness of our method by comparing it with a least-squares-based method [20] that assumes that all detected sphere centers are accurate.

The remainder of this paper is organized as follows. Section 2 reviews related work. Section 3 presents our proposed methods for circle detection and extrinsic calibration of multiple RGB-D cameras. Section 4 shows experimental results on the accuracy of the detected circle centers and the estimated extrinsic parameters. Finally, Section 5 concludes the paper.

## 2. Related Work

The depth camera equipped in an RGB-D camera is typically a Time-of-Flight (ToF) camera [6] or a Structured-Light (SL) 3D camera [5,7]. Both depth cameras illuminate the scene with Infrared (IR) light and receive the reflected light with an IR camera (or a two-dimensional array of IR sensors). The depth cameras all suffer from irregular noise and systematic errors. Early research works focused on modeling and reducing noise and errors [24,25,26,27].

Many researchers have developed methods for measuring the systematic bias existing in the depths acquired with ToF or SL 3D cameras [28,29,30,31]. Because the bias depends on various factors such as pixel location, IR intensity, and measured distance, it is difficult to model the bias without an external distance measuring device. Thanks to Zhang’s calibration method [8], a color camera is ready to play the role [28,29,30,31]. Once the color camera is calibrated by using a planar checkerboard [8], it is possible to estimate the plane parameters of the checkerboard in an image. Assuming that the extrinsic parameters are given between the color camera and the depth camera, it is possible to transform the plane parameters to the depth camera’s coordinate system. The planar depth is then subtracted from the measured depth to compute the per-pixel biases. Based on the fact that the extrinsic calibration and systematic bias modeling are tightly-coupled problems, Basso et al. [31] proposed an optimization framework to solve both problems simultaneously.

Kim et al. [28] and Yang et al. [32] applied Zhang’s method [8] to color and IR images of the checkerboard so that the color camera and the depth camera will be extrinsically calibrated. To maximize the visibility of the calibration object in the IR images, Jung et al. [30] proposed to use a specially-designed board with round holes. For extrinsic calibration of a camera and a laser range finder, Ha [33] suggested using a checkerboard with a triangular hole, which simplifies establishing 3D point correspondences across the sensors. Herrera et al. [29] proposed an IR image-free approach based on a planarity constraint that the transformed plane should coincide with the plane of the depth image. This method uses the checkerboard pattern on a large planar surface to establish 3D plane correspondences across the cameras. Fernández-Moral et al. [34] also proposed to use 3D plane correspondences for extrinsic calibration of a set of depth cameras. Perez-Yus et al. [35] proposed to use line correspondences for extrinsic calibration of a color and depth camera pair or a system of multiple RGB-D cameras. In their method, the line correspondences can be given manually or randomly. Perez-Yus et al. [35] provided an experimental result showing the effect of the randomly-given correspondences. This paper focuses on the extrinsic calibration between different RGB-D cameras, assuming that the individual RGB-D cameras have been fully calibrated.

It is possible to apply Zhang’s method [8], Herrera et al.’s method [29], or Fernández-Moral et al.’s method [34] to extrinsic calibration of different depth cameras. If we use the IR images of the checkerboard [28,32], the extrinsic calibration between depth cameras is the same as for color cameras [8]. In this case, because a pair of IR images provides correspondences on a single plane, we need to acquire several pairs of IR images, placing the checkerboard differently. Without using the IR images, the extrinsic parameters between depth cameras can be estimated by acquiring depth images of at least three different poses of a planar board [29,34]. The same side of the checkerboard or the planar board is, however, hardly viewed simultaneously by widely-separated RGB-D cameras. Therefore, the cameras need to be densely distributed so that adjacent cameras have a large common field of view.

Spheres have been used to calibrate color cameras, as well as RGB-D cameras [12,13,14,15,16,17,18,19,20,21]. Agrawal et al. [12] and Zhang et al. [13] proposed methods for both intrinsic and extrinsic calibration of color cameras. The methods use three or more images of a sphere at different places. For extrinsic calibration, the methods estimate the 3D positions of the spherical centers from the projections of the occluding contours of the sphere. The 3D positions are then used for 3D point registration between two cameras. For the lack of direct 3D measurements on the sphere, the accuracy of the methods is highly affected by the ellipse fitting of the sphere projections. For robust extrinsic calibration to ellipse fitting error, Guan et al. [14] used Zhang’s method [8] to recover the intrinsic parameters assuming that the sphere centers project to the centroids of the sphere projections. The depth values of the sphere centers were then estimated by using the area of the projections.

To our best knowledge, Shen et al. [15] first used a sphere as a calibration object for a wide-baseline RGB-D camera network. They proposed methods for RGB-and-depth calibration and depth-and-depth calibration. Later, Su et al. [20] extended their former work [15] to use nonrigid extrinsic parameters to reduce errors in the fused point cloud. Ruan and Huber [16] proposed an optimization method for estimating the extrinsic parameters and simultaneously correcting the sphere centers. Staranovicz et al. [17] showed that an RGB-D camera can be both intrinsically and extrinsically calibrated using a sphere. Later, they extended their work to extrinsic calibration of multiple RGB-D cameras [18].

To simplify the detection of spherical objects in color images, previous approaches used a lighted sphere [12,14] or a sphere painted in a unique color [13,15,16,17,18,19,20]. The approaches apply a simple threshold [12,14] or background subtraction algorithm [15,16,17,18,20] to reduce the search regions for the spherical object. Shen et al. [15] and Su et al. [20] used a color-based probability model to detect projections of the sphere. Staranovicz et al. [17,18] used the circular Hough transform [36] to find circular shapes in the segmented foreground regions. Under controlled lighting conditions, background subtraction and color-based detection will give accurate results. However, in uncontrolled environments, the lighting may not be uniform, and it may be difficult to build a background model. In addition, the background may contain objects of similar color to the sphere. In our former work [21], the experimental results showed that the color-based probability model often fails under different lighting conditions.

The estimated sphere centers may be inaccurate for several reasons. For example, the RGB-D cameras may be asynchronous [19], and the sphere detection algorithms [15,16,17,18,20] can detect the wrong objects. Shen et al. [15] and Su et al. [20] synchronized their capture system using the network time protocol. Lee et al. [19] proposed an algorithm to compensate for the synchronization error in the estimated sphere centers. Several methods [15,16,20] do not assume false detection, while other methods [18,19] apply RANSAC [22,37] to reject outliers in pairwise camera pose estimation.

To capture multiple color and depth image pairs simultaneously, we connected all RGB-D cameras to a single computer and used multiple threads to invoke simultaneously the capture functions. However, this does not guarantee perfect synchronization. Our optimization-based framework relies on robust loss functions and provides accurate calibration results without explicitly detecting or rejecting outliers. With a large number of images, the proposed method can improve calibration accuracy under incomplete synchronization.

## 3. Proposed Method

**Figure 1:**
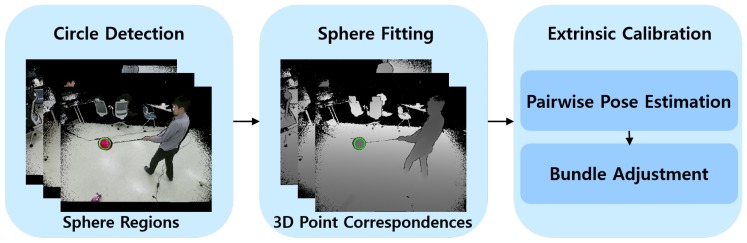
Proposed method for extrinsic calibration of multiple RGB-D cameras. The green circles represent detected sphere regions. Refer to the text for more detail.

Given *M* RGB-D cameras and *N* color and depth image pairs per camera, our circle detection algorithm finds sphere regions in the M×N color images. Sphere centers are then estimated in the corresponding regions of the depth images. The M×N sphere centers are used as 3D point correspondences across the depth cameras. The pairwise poses between different depth cameras are estimated using the correspondences. The 3D positions of the centers, as well as the poses, are refined by bundle adjustment [38]. Figure 1 summarizes the proposed method.

### 3.1. Robust Estimation

Our proposed method heavily relies on robust loss functions, which clip the magnitude of error *e* to a fixed value τ. In each stage of our method, *e* is differently defined. For example, *e* can be the distance between pixels, between 3D points, or between lengths, with physical units in pixels or centimeters. If we use absolute errors, the robust loss function $\rho_{\tau }\left(|e|\right)$ is defined as:

(1) $$\rho_{\tau }\left(|e|\right)=\left\{\begin{array}{cc} \left|e\right| & \mathrm{if}\;|e|<\tau ,\\ \tau & \mathrm{otherwise}, \end{array}\right.$$

where τ is the error-clipping value associated with ρ, and τ’s unit also depends on its application. Table 1 summarizes the values and units of τ used in this paper. Analogously, if we use square errors, $\rho_{\tau^{2}}\left(e^{2}\right)$ is defined as:

(2) $$\rho_{\tau^{2}}\left(e^{2}\right)=\left\{\begin{array}{cc} e^{2} & \mathrm{if}\;e^{2}<\tau^{2},\\ \tau^{2} & \mathrm{otherwise}. \end{array}\right.$$

In robust estimation, the cost function is usually defined as the sum of the robust loss functions. Because the robust loss functions clip the magnitude of error, they reduce the effect of outliers on the cost.

We also frequently used the M-estimator Sample Consensus (MSAC) framework [23], which provides a systematic way to use the robust loss function for model parameter estimation. MSAC iteratively selects random samples of the minimum number of elements required to compute model parameter p. In our method, the model ranges from a circle to a rigid transformation between cameras. The elements are usually points or point pairs, and their type, as well as their minimum number vary with the model. For example, if the model is a circle, the parameter vector p consists of the center and radius of the circle, and we need at least three points to compute p.

In MSAC, a robust cost function J(p) is evaluated for each p so as to find the best $p^{★}$ minimizing J(p). For example, J(p) can be defined as:

(3) $$J\left(p\right)=\sum_{i}\rho_{\tau^{2}}\left(e_{i}^{2}\right),$$

where $e_{i}$ is the error computed from the *i*th element.

The MSAC procedure used in this paper is summarized in Algorithm 1. In Algorithm 1, $N_{S}$ is the total number of samples, and more discussion on the setting of $N_{S}$ will be given in Section 3.6. J(p) can be minimized further by applying nonlinear optimization algorithms such as the Levenberg–Marquardt algorithm [39,40]; however, we skip this step unless otherwise mentioned.

**Algorithm 1:** General MSAC procedure. **Result**: $p^{★}$ minimizing J(p)
 $J_{\min}=$ the maximum value of the data type of J(p);  $p^{★}=$ an arbitrary value or vector;  
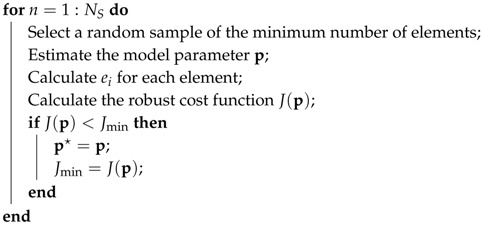


### 3.2. Multi-Cue Based Circle Detection

Because we assume that the lighting may vary across images, we represent color in a space that is less affected by lighting changes. For this purpose, we transform RGB values to the $CIE-La^{*}b^{*}$ space in order to use only $a^{*}$ and $b^{*}$ components, which are less affected by lighting changes at the expense of reduced distinctiveness.

We assume that the spherical object is monochromatic. Let us denote the mean color of the spherical object as $a_{m}=\left(a_{m}^{*},b_{m}^{*}\right)$, which can be computed from a training set captured at different places under different lighting conditions [21]. Given a color image, we can find the pixel with color $a_{d}^{\left(1\right)}$ that is the most distant from $a_{m}$. The pixels in the image are then divided into two disjoint sets $S_{d}^{\left(1\right)}$ and $S_{m}^{\left(1\right)}$ according to their color distances from $a_{m}$ and $a_{d}^{\left(1\right)}$. If a pixel’s color is closer to $a_{m}$, it belongs to $S_{m}^{\left(1\right)}$, and otherwise, $S_{d}^{\left(1\right)}$. In this manner, we can recursively divide $S_{m}^{\left(k-1\right)}$ into two disjoint sets $S_{m}^{\left(k\right)}$ and $S_{d}^{\left(k\right)}$ by finding $a_{d}^{\left(k\right)}$ in $S_{m}^{\left(k-1\right)}$ with the maximum value of $∥a_{d}^{\left(k\right)}-a_{m}∥$.

**Figure 2:**
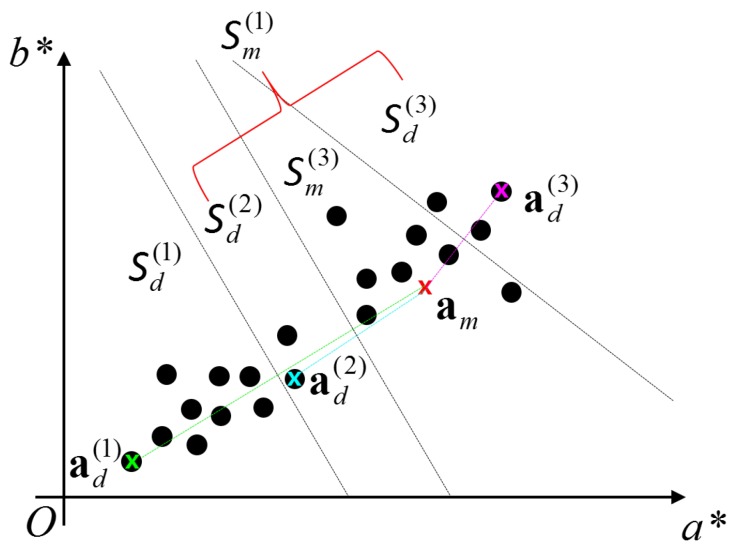
Color-based pixel clustering. The black circles represent pixel colors. The most distant color from $a_{m}$ is $a_{d}^{\left(1\right)}$, and the pixels are divided into two disjoint sets $S_{d}^{\left(1\right)}$ and $S_{m}^{\left(1\right)}$, which is the union of $S_{m}^{\left(3\right)}$, $S_{d}^{\left(3\right)}$, and $S_{d}^{\left(2\right)}$. The black dotted lines represent the boundary between different sets of pixels. Refer to the text for more detail. Best viewed in color.

**Figure 3:**
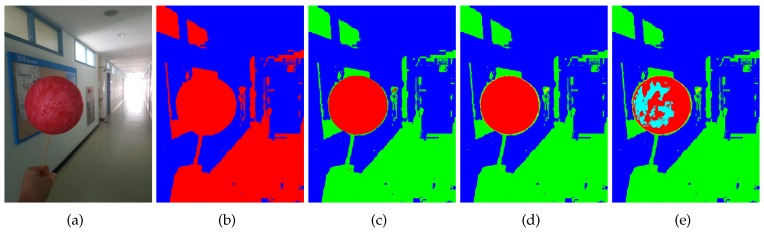
Color-based pixel clustering example. (**a**) Input color image. (**b**) $S_{m}^{\left(1\right)}$ and $S_{d}^{\left(1\right)}$ differently color-coded. $S_{m}^{\left(1\right)}$ and $S_{d}^{\left(1\right)}$ are colored in red and blue, respectively. (**c**) $S_{m}^{\left(2\right)}$, $S_{d}^{\left(1\right)}$, and $S_{d}^{\left(2\right)}$ colored in red, blue, and green, respectively. (**d**) $S_{m}^{\left(3\right)}$, $S_{d}^{\left(1\right)}$, and $S_{d}^{\left(2\right)}$, $S_{d}^{\left(3\right)}$ colored in red, blue, green, and yellow, respectively. (**e**) $S_{m}^{\left(4\right)}$, $S_{d}^{\left(1\right)}$, $S_{d}^{\left(2\right)}$, $S_{d}^{\left(3\right)}$, and $S_{d}^{\left(4\right)}$ colored in red, blue, green, yellow, and cyan, respectively.

Figure 2 illustrates the color-based pixel clustering procedure. As shown in the figure, the pixels near the mean color are recursively separated from farther pixels. The recursive procedure lets us have a good chance of detecting the sphere region as shown in Figure 3.

**Figure 4:**
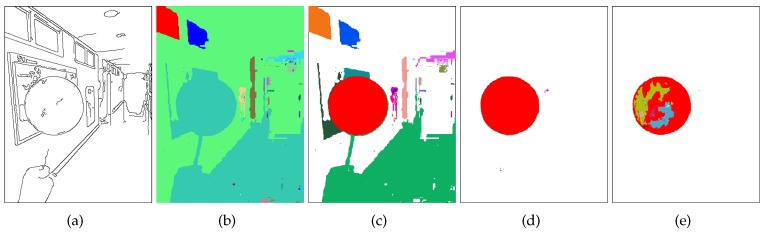
Edge pixels and connected components. (**a**) Edge image of Figure 3a. (**b**) Connected regions detected in $S_{m}^{\left(1\right)}$ and $S_{d}^{\left(1\right)}$, differently color-coded. (**c**) Connected regions detected in $S_{m}^{\left(2\right)}$ and $S_{d}^{\left(2\right)}$. (**d**) Connected regions detected in $S_{m}^{\left(3\right)}$ and $S_{d}^{\left(3\right)}$. (**e**) Connected regions detected in $S_{m}^{\left(4\right)}$ and $S_{d}^{\left(4\right)}$.

Due to the varying lighting condition, however, there is no guarantee that the projected region of the spherical object will be always composed of pixels in $S_{m}^{\left(k\right)}$. Therefore, we detect all the connected regions [41] in $S_{m}^{\left(k\right)}$ and $S_{d}^{\left(k\right)}$ for all k=1,…,K, where K=30 throughout this paper. In the meantime, if $∥a_{d}^{\left(k\right)}-a_{m}∥$ is less than $d_{\min}$ (in this paper, 10), we stop the pixel clustering procedure. Figure 4 shows examples of the connected regions recursively detected in $S_{m}^{\left(k\right)}$ and $S_{d}^{\left(k\right)}$.

We assume that at least one of the connected regions is at least partially the projection of the spherical object and that the shape of the region is circular. For a connected region to be circular, the region’s boundary pixels should be near the circumference. If there are edge pixels [42] near the circular boundary pixels, the edge pixels are strong evidence for the existence of the circle. Therefore, we use both boundary pixels and edge pixels to detect circular regions.

To estimate the center $c=(c_{x},c_{y})$ and the radius *r* of a connected region, we use MSAC [23]. Given boundary pixel locations $x_{i}=\left(x_{i},y_{i}\right)$ for $i=1,...,N_{CF}$, we find c and *r* minimizing the following cost function.

(4) $$J_{CF}\left(c,r\right)=\sum_{i=1}^{N_{CF}}\rho_{\tau_{CF}}(|∥x_{i}-c∥-r|),$$

where $\tau_{CF}=3$ pixels throughout this paper.

Given three different boundary pixels, it is straightforward to compute a pair of c and *r*. Without loss of generality, let us denote such three pixel locations as $x_{1}=\left(x_{1},y_{1}\right)$, $x_{2}=\left(x_{2},y_{2}\right)$, and $x_{3}=\left(x_{3},y_{3}\right)$. For the three pixels to be on the circumference, the following linear equation should hold:

(5) $$\left(x_{i}-x_{1}\right)\cdot c_{x}+\left(y_{i}-y_{1}\right)\cdot c_{y}=0.5\cdot \left(x_{i}^{2}-x_{1}^{2}+y_{i}^{2}-y_{1}^{2}\right)$$

for i=2,3. c is attained by solving the linear equations. Given c, it is simple to compute *r*, which is given by:

(6) $$r=\sqrt{{\left(x_{i}-c_{x}\right)}^{2}+{\left(y_{i}-c_{y}\right)}^{2}}$$

for any i∈{1,2,3}.

We randomly draw samples of three boundary pixels $N_{S}$ times to calculate $N_{S}$ different pairs of c and *r*, where $N_{S}=1000$. Then, the pair minimizing Equation (4) is chosen as the solution.

For the sake of efficiency, circles are fitted only to connected regions such that at least $P_{\min}$% (in this paper 10%) of their pixels are from $S_{m}^{\left(1\right)}$. In addition, we reject connected regions that are too large or too small based on the number of pixels in the regions. For a circle to be fitted to a connected region, the number of pixels should be greater than $\pi r_{min}^{2}$ and less than $\pi r_{max}^{2}$, where $r_{min}$ is set to 10 pixels and $r_{max}$ is half of the image width or height. If there is no such connected region, no circle is detected by the proposed method.

**Figure 5:**
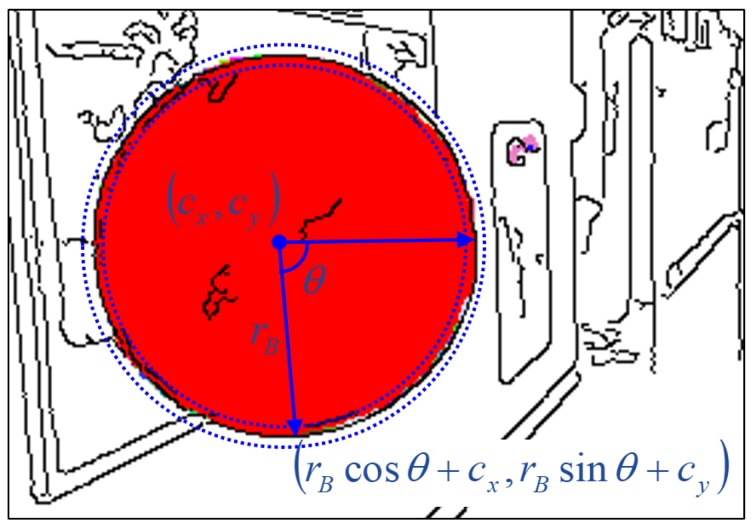
Multi-cue-based circle detection. Edge pixels in Figure 4a are drawn in black on the connected regions in Figure 4d. The blue dashed circles represent the search range from $r-\tau_{MC}$–$r+\tau_{MC}$ for region boundary pixels. Refer to the text for more detail.

For all the fitted circles, we calculate the following multi-cue-based cost $J_{MC}\left(c,r\right)$.

(7) $$J_{MC}\left(c,r\right)=\frac{1}{\tau_{MC}}\sum_{\theta =0^{\circ }}^{359^{\circ }}\min_{r_{B}}(\rho_{\tau_{MC}}\left(\right|r_{B}-r|)+\rho_{\tau_{MC}}\left(d_{E}\left(\theta ,r_{B}\right)\right)),$$

where θ is a quantized angle ranging from $0^{\circ }$–$359^{\circ }$ with $1^{\circ }$ resolution and $r_{B}$ is increased from $r-\tau_{MC}$–$r+\tau_{MC}$ to find a boundary pixel within a one-pixel range from $\left(r_{B}\cos\theta +c_{x},r_{B}\sin\theta +c_{y}\right)$. Here, $\tau_{MC}$ is set to the smaller value between $\tau_{CF}$ and 0.1r to discourage small nonexistent circles from being detected. $d_{E}\left(\theta ,r_{B}\right)$ is the distance from the boundary pixel to its nearest edge pixel. The boundary pixel is not always unique for a pair of θ and $r_{B}$; this is why we compute the minimum value in Equation (7). For some θ, there may be no boundary pixel within the range of $r_{B}$. In this case, we set $\rho_{\tau_{MC}}\left(\right|r_{B}-r\left|\right)+\rho_{\tau_{MC}}\left(d_{E}\left(\theta ,r_{B}\right)\right)$ to $2\tau_{MC}$. Figure 5 illustrates an example of a boundary pixel for a pair of θ and $r_{B}$. As shown in the figure, both boundary pixels and edge pixels tend to be near the circumference if the region is circular. The proposed circle detection algorithm is summarized in Algorithm 2.

**Algorithm 2:** Proposed circle detection algorithm. **Result**: $\left(c^{★},r^{★}\right)$ minimizing $J_{MC}\left(c,r\right)$
 i=1;  $S_{m}^{\left(0\right)}=$ the set of the entire pixels in the input image;  
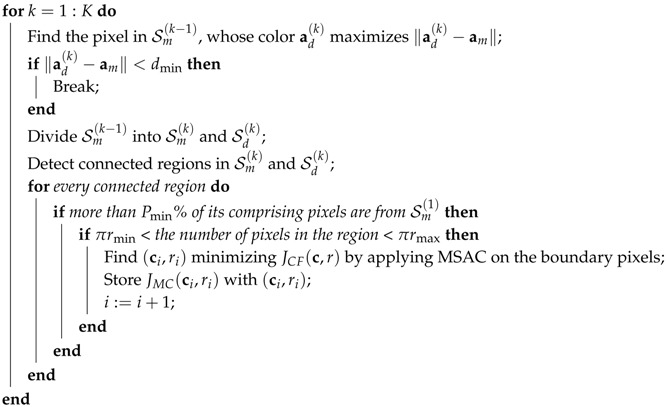

 Sort ${\left\{J_{MC}\left(c_{j},r_{j}\right)\right\}}_{j=1}^{i-1}$ in ascending order;  $\left(c^{★},r^{★}\right)=\left(c_{1},r_{1}\right)$ with the least $J_{MC}\left(c,r\right)$ in the sorted set.

### 3.3. Sphere Fitting

Given a circular region in a color image, it is possible to read out 3D point measurements $X_{i}=\left(X_{i},Y_{i},Z_{i}\right)$ for $i=1,\ldots ,N_{SF}$ in the corresponding region of the depth image because we assume that our individual RGB-D cameras have been fully calibrated. Given the 3D points, the MSAC procedure [23] is applied to estimate the sphere center $C=(C_{X},C_{Y},C_{Z})$. We minimize:

(8) $$J_{SF}\left(C,R\right)=\sum_{i=1}^{N_{SF}}\rho_{\tau_{SF}}(|∥X_{i}-C∥-R|),$$

where $\tau_{SF}$ is set to 2 cm.

Given four 3D points on the sphere, for example $X_{1}$, $X_{2}$, $X_{3}$, and $X_{4}$, without loss of generality, we can compute C by solving the following linear equation:

(9) $$\left(X_{i}-X_{1}\right)\cdot C_{X}+\left(Y_{i}-Y_{1}\right)\cdot C_{Y}+\left(Z_{i}-Z_{1}\right)\cdot C_{Z}=0.5\cdot \left(X_{i}^{2}-X_{1}^{2}+Y_{i}^{2}-Y_{1}^{2}+Z_{i}^{2}-Z_{1}^{2}\right)$$

for i=2,3,4. Given C, it is simple to compute *R*, which is given by:

(10) $$R=\sqrt{{\left(X_{i}-C_{X}\right)}^{2}+{\left(Y_{i}-C_{Y}\right)}^{2}+{\left(Z_{i}-C_{Z}\right)}^{2}}$$

for any i∈{1,2,3,4}. We set the number of random samples $N_{S}$ to 10,000 to increase the probability of drawing at least one sample of four accurate 3D points in the MSAC procedure.

### 3.4. Pairwise Pose Estimation

Now, we have sphere centers $C_{i}^{\left(q\right)}$ for q=1,…,M and i=1,…,N. Let us denote the visibility of $C_{i}^{\left(q\right)}$ as $v_{i}^{\left(q\right)}$, which is one if $C_{i}^{\left(q\right)}$ is visible (or has been detected by our algorithm), and otherwise zero.

Without loss of generality, let us assume that the reference depth camera is the first one. For each pair of depth cameras 1 and *q*, we apply MSAC [23] to compute the rigid transformation from Depth Camera 1 to depth camera *q* such that:

(11) $$C_{i}^{\left(q\right)}=R_{q}C_{i}^{\left(1\right)}+T_{q},$$

where $R_{q}$ is the 3×3 rotation matrix and $T_{q}$ is the 3D translation vector of the rigid transformation.

Given four image frames (or four visible sphere center pairs), we can compute the rigid transformation. For more detail on the computation, please refer to [22]. We randomly sample four image frames iteratively to find the rigid transformation minimizing:

(12) $$J_{RT}\left(R_{q},T_{q}\right)=\sum_{i=1}^{N}v_{i}^{\left(q\right)}\cdot v_{i}^{\left(1\right)}\cdot \rho_{\tau_{RT}^{2}}(∥C_{i}^{\left(q\right)}-R_{q}C_{i}^{\left(1\right)}-T_{q}{∥}^{2}).$$

For this task, we set the number of random samples $N_{S}$ to 10,000 and $\tau_{RT}$ to $2\sqrt{3}$ cm.

In this paper, the pairwise pose is computed between the reference depth camera and each of the remaining depth cameras. In practice, the pairwise pose may have to be computed between adjacent depth cameras due to the lack of corresponding points. In this case, $R_{q}$ and $T_{q}$ can be calculated from the poses between adjacent depth cameras.

### 3.5. Bundle Adjustment

Our Bundle Adjustment (BA) procedure refines ${\left\{R_{q},T_{q}\right\}}_{q=2}^{M}$ and sphere centers ${\left\{C_{i}\right\}}_{i=1}^{N}$ in the reference camera’s coordinate system. $C_{i}$ is initialized to the median of ${\tilde{C}}_{i}^{\left(q\right)}$, where ${\tilde{C}}_{i}^{\left(q\right)}=R_{q}^{T}\left(C_{i}^{\left(q\right)}-T_{q}\right)$. Our cost function for BA is defined as:

(13) $$\begin{array}{cc} & J_{BA}\left({\left\{R_{q},T_{q}\right\}}_{q=2}^{M},{\left\{C_{i}\right\}}_{i=1}^{N}\right)\\ = & \sum_{q=1}^{M}\sum_{i=1}^{N}v_{i}^{\left(q\right)}\cdot \left(\rho_{\tau_{BA}^{2}}\left({\left(C_{i,X}^{\left(q\right)}-{\hat{C}}_{i,X}^{\left(q\right)}\right)}^{2}\right)+\rho_{\tau_{BA}^{2}}\left({\left(C_{i,Y}^{\left(q\right)}-{\hat{C}}_{i,Y}^{\left(q\right)}\right)}^{2}\right)+\rho_{\tau_{BA}^{2}}\left({\left(C_{i,Z}^{\left(q\right)}-{\hat{C}}_{i,Z}^{\left(q\right)}\right)}^{2}\right)\right), \end{array}$$

where ${\hat{C}}_{i}^{\left(q\right)}={\left({\hat{C}}_{i,X}^{\left(q\right)},{\hat{C}}_{i,Y}^{\left(q\right)},{\hat{C}}_{i,Z}^{\left(q\right)}\right)}^{T}=R_{q}C_{i}+T_{q}$, and $\tau_{BA}$ is set to 2 cm. We note that because the first depth camera is the reference one, $R_{1}$ and $T_{1}$ are fixed to the identity matrix and the zero vector, respectively.

The Levenberg–Marquardt algorithm [39,40] can minimize the sum of squares. The proposed method uses a robust loss function, but $J_{BA}$ is still the sum of the squares of the truncated differences. Therefore, we use the Levenberg–Marquardt algorithm [39,40] to minimize the cost. Algorithm 3 summarizes the proposed extrinsic calibration method.

**Algorithm 3:** Proposed extrinsic calibration algorithm.**Result**: ${\left\{R_{q}^{★},T_{q}^{★}\right\}}_{q=2}^{M}$, ${\left\{C_{i}^{★}\right\}}_{i=1}^{N}$ minimizing $J_{BA}\left({\left\{R_{q},T_{q}\right\}}_{q=2}^{M},{\left\{C_{i}\right\}}_{i=1}^{N}\right)$
 
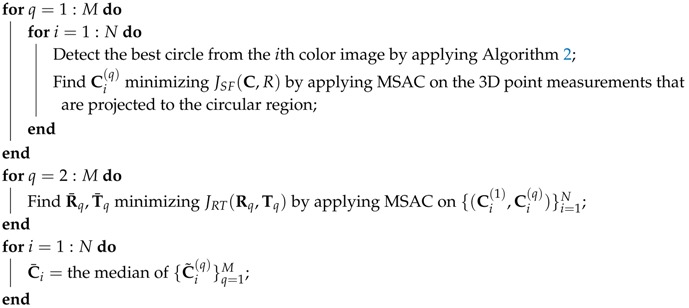

 Apply the Levenberg–Marquardt algorithm to find ${\left\{R_{q}^{★},T_{q}^{★}\right\}}_{q=2}^{M}$, ${\left\{C_{i}^{★}\right\}}_{i=1}^{N}$ minimizing   $J_{BA}\left({\left\{R_{q},T_{q}\right\}}_{q=2}^{M},{\left\{C_{i}\right\}}_{i=1}^{N}\right)$, with ${\left\{{\bar{R}}_{q},{\bar{T}}_{q}\right\}}_{q=2}^{M}$, ${\left\{{\bar{C}}_{i}\right\}}_{i=1}^{N}$ as the initial solution;

### 3.6. Discussion on Parameter Settings

The proposed method depends on more than ten parameters, which are summarized in Table 1. The proposed method uses the robust loss function ρ in every stage, and ρ’s associated clipping value τ varies with the application. For example, in circle fitting, τ is denoted by $\tau_{CF}$, and its unit is in pixels. We also use ρ with $\tau_{MC}$ to compute the multi-cue-based cost $J_{MC}$ for every fitted circle. The values of $\tau_{SF}$, $\tau_{RT}$, and $\tau_{BA}$ depend on the depth noise, whose magnitude is reported as about 1 cm for Kinect v2 cameras [43]. We recommend the users try several integer multiples of the noise magnitude to set the values of $\tau_{SF}$ and $\tau_{BA}$. Once $\tau_{BA}$ has been set, it is reasonable to set $\tau_{RT}$ to $\sqrt{3}\tau_{BA}$, considering the forms of the cost functions $J_{RT}$ and $J_{BA}$.

$N_{S}$ is the total number of samples in MSAC, which also varies with the application. $N_{S}$ is dependent on the proportion *w* of inliers and the minimum number *s* of elements for generating a hypothesis [22]:

(14) $$N_{S}=\frac{\log(0.01)}{\log(1-w^{s})}.$$

Using $N_{S}$ in Equation (14), it is possible to compute a valid solution with 99% of probability. To increase the probability, we can decrease 0.01 to a smaller value. In circle fitting, we need at least three boundary pixels (s=3) to compute the circle center and radius. We need at least four 3D surface points (s=4) to compute the sphere center and radius. In pairwise pose estimation, we also need at least four sphere-center pairs (s=4) to compute the rotation and translation between cameras. According to Hartley and Zisserman [22], the values can be set to a number around 100 when *w* is about 0.5. In practice, however, it is hard to know *w*, so we set $N_{S}$ to 1000 or 10,000 to avoid failure in the presence of only a handful of inliers.

The mean sphere color $a_{m}$ is the most important parameter that must be learned if the spherical object is differently painted. The mean sphere color in Table 1 has been computed from the 30 training images in Kwon et al.’s dataset [21]. $P_{\min}$ controls the flexibility of the proposed method: if $P_{\min}$ is high, the circle detection method tends to detect circles whose color is different from $a_{m}$. We set $P_{\min}$ to a low value (10%) to maximize the robustness to lighting changes. Although we use different spherical objects in the next section, we do not tune the parameters of the proposed method to test its robustness to changes in the objects. We use the same parameters throughout this paper.

## 4. Experiments

In this section, we provide experimental results on the accuracy and robustness of the proposed method. The first subsection shows the experimental results of the proposed circle detection method on datasets with cluttered backgrounds and different lighting conditions. The second subsection shows the experimental results of the proposed extrinsic calibration method on datasets with inaccurate sphere centers.

### 4.1. Circle Detection Results

To show the effectiveness of the proposed circle detection method, we used three different datasets. The first dataset (styrofoam ball set) is from our former work [21]. The dataset consists of 138 images of a red styrofoam ball acquired at different places under different lighting conditions. The size of the images is typically 360×480 pixels. The dataset contains the manually-recorded center locations of the projected sphere regions. Some images include a blue sphere; however, its projected locations are not included in the dataset. Of these 138 images, we used 108 test images in the experiments. The mean color of the sphere was computed from the remaining 30 training images.

The second and third datasets were newly collected to show the robustness of the proposed method to changes in the calibration object. The second dataset (gym ball set) and the third dataset (basketball set) consist of 131 gym ball images and 131 basketball images, respectively. The images were acquired at similar places to the styrofoam ball set. The gym ball is monochromatic, and its color is very similar to the styrofoam ball. By contrast, the basketball has a pattern, and its color is not like the styrofoam ball. The new datasets do not include the blue sphere. We note that the parameters of the proposed method and Kwon et al.’s method [21] were not adjusted for the new datasets. This means that the mean color of the styrofoam ball is consistently used throughout this paper.

The proposed method was compared with three existing methods. The first method was our former method using the color of the sphere and a template-based search [21]. The second method was the OpenCV implementation [9] of the Circular Hough Transform (CHT) [36]. Finally, the third method (EDCircles) [44] was one of the top performers in a recent evaluation paper [45], whose source code is publicly available online. We used the authors’ source code with their default parameters. EDCircles fits an ellipse to a detected circle with high eccentricity. In this case, we use the circle’s original center for the evaluation. We also note that EDCircles rejects false circles and sometimes returns no circle.

The circles detected by our method can be sorted in ascending order of Equation (7) to find the best circle in an image. Similarly, the circles detected by the three existing methods can be sorted in descending order of their own circle-ness. Therefore, we can compare the accuracy of the best circles, as well as the top *m* circles.

The first two methods [21,36], as well as our method use the Canny edge detector [42]. We used the same Canny edge parameters for all three methods. In addition, we used the same number of MSAC iterations, $N_{S}=1000$, to refine the results of [21]. Finally, we set the minimum and the maximum radius of the methods to the same values: the minimum is set to 10 pixels, and the maximum is set to half of the image width or height.

**Figure 6:**
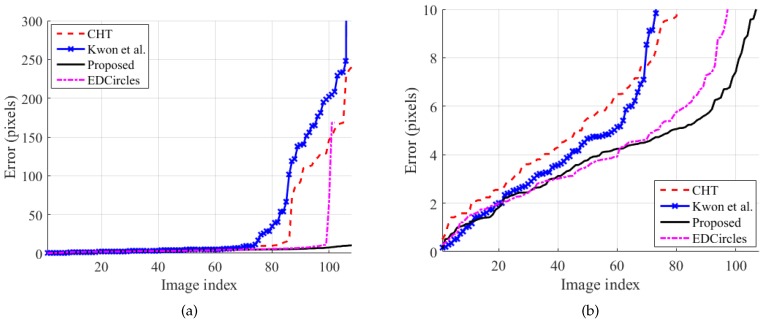
Circle detection accuracy (styrofoam ball set). The error is the distance from the best circle center detected by a method to its manually-recorded ground-truth center. For each method, the error has been sorted in ascending order, so the image indices do not match across the methods. (**b**) has been scaled from (**a**) to show the number of detected circles with low errors. Best viewed in color. CHT, Circular Hough Transform.

Figure 6 shows the accuracy of the methods on the styrofoam ball set. The error in the figure is the distance from the best circle center detected by a method to its manually-recorded ground-truth center. Considering that the manually-recorded center locations will have errors from the human operator, the proposed method provided accurate results for all test images with a maximum error of 10 pixels. EDCircles gave similar results to the proposed method for most of the images. In Figure 6a, it can be seen that the horizontal length of the EDCircles’ curve is shorter than the others because of its false rejection ability.

**Figure 7:**
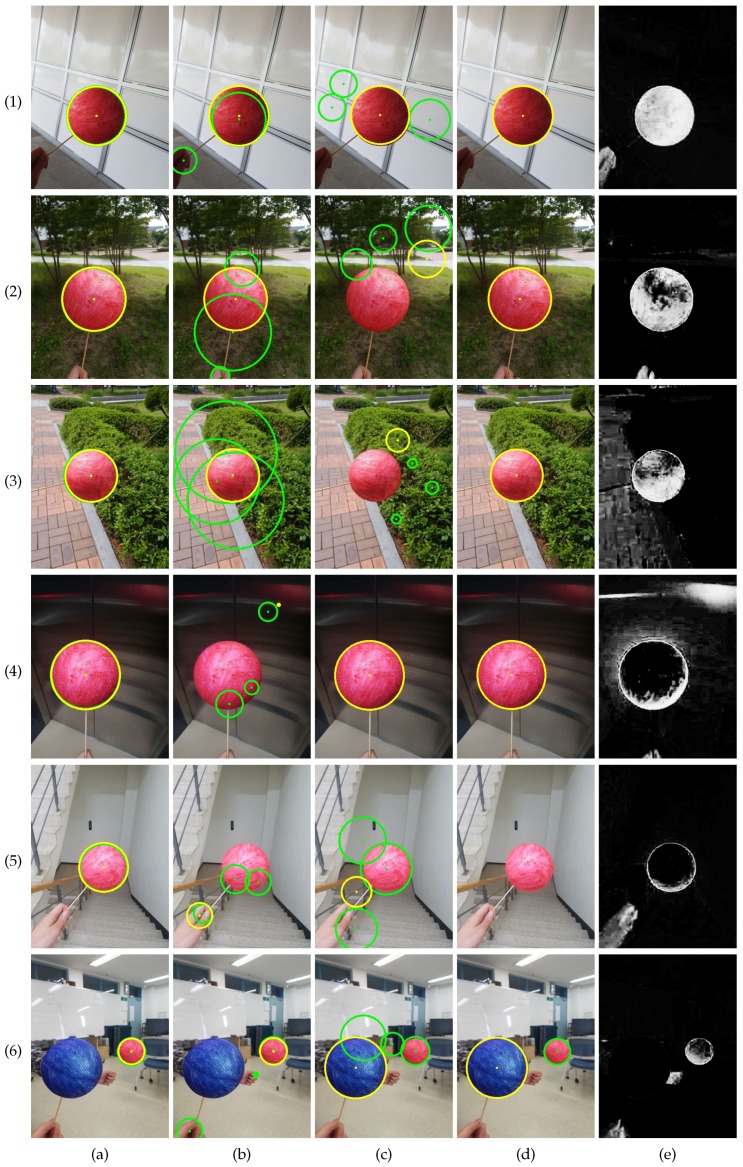
Top four circles detected by the methods (styrofoam ball set). (**a**) Proposed method. (**b**) Kwon et al. [21]. (**c**) CHT [36]. (**d**) EDCircles [44]. (**e**) Color probability images [21]. From (**a**–**d**), the best circles are drawn in yellow, while the remaining circles are drawn in green. Some circles have similar centers and radii in (**a**,**b**), so the numbers of circles in the images may seem less than four. Best viewed in color.

Figure 7 shows sample results attained by the four methods. Figure 7(1) shows an image for which all four methods provide good results. The background is simple, and the color model in Figure 7(1)e accurately localizes the sphere. Figure 7(2) and (3) show images with cluttered background. CHT [36] failed to attain accurate results on the images, while EDCircles [44] detected the circles accurately. This is because EDCircles found circles in long connected arcs that are rarely detected in the cluttered background. Figure 7(4) and (5) show images for which the color model in [21] failed to assign a high probability to the sphere. Consequently, Kwon et al.’s method [21] failed to attain accurate results. It is interesting to notice that EDCircles had rejected the circle in Figure 7(5). Finally, Figure 7(6) shows an image containing a large blue sphere. Because CHT and EDCircles do not use color information, they found the blue sphere preferentially.

**Figure 8:**
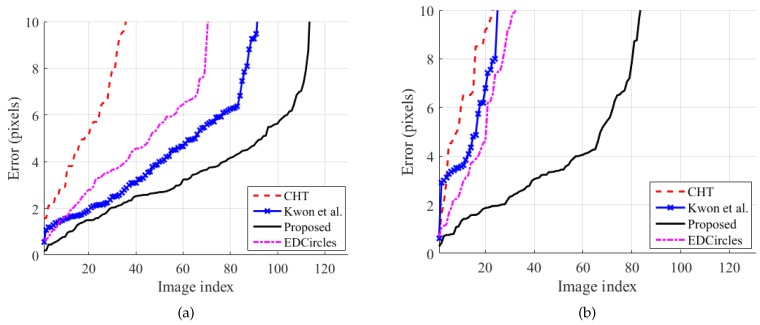
Circle detection accuracy. (**a**) Gym ball set. (**b**) Basketball set. The error is the distance from the best circle center detected by a method to its manually-recorded ground-truth center. For each method, the error has been sorted in ascending order, so the image indices do not match across the methods. Best viewed in color.

Figure 8 shows the accuracy of the methods on the gym ball set and the basketball set. The proposed method outperforms the existing methods on the two new datasets. On the gym ball set, Kwon et al.’s method outperformed CHT and EDCircles. We conjecture that the similarity in color between the gym ball and the styrofoam ball is the main reason for the better performance. This conjecture is supported by the performance degradation of Kwon et al.’s method on the basketball set, as shown in Figure 8b. Because the proposed method relies less on color information, it suffered less performance degradation than Kwon et al.’s method. It is also surprising that the basketball was detected in many images by the proposed method in spite of the assumption that the sphere was monochromatic.

**Figure 9:**
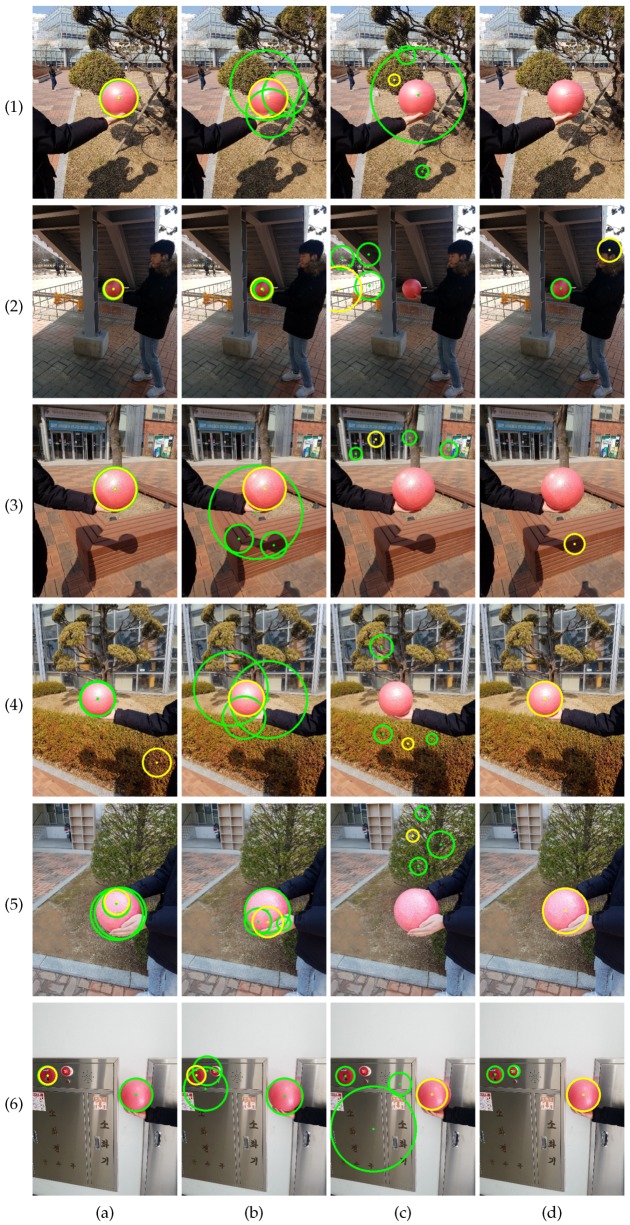
Top four circles detected by the methods (gym ball set). (**a**) Proposed method. (**b**) Kwon et al. [21]. (**c**) CHT [36]. (**d**) EDCircles [44]. From (**a**–**d**), the best circles are drawn in yellow, while the remaining circles are drawn in green. Some circles have similar centers and radii in (**a**) and (**b**), so the numbers of circles in the images may seem less than four. Best viewed in color.

**Figure 10:**
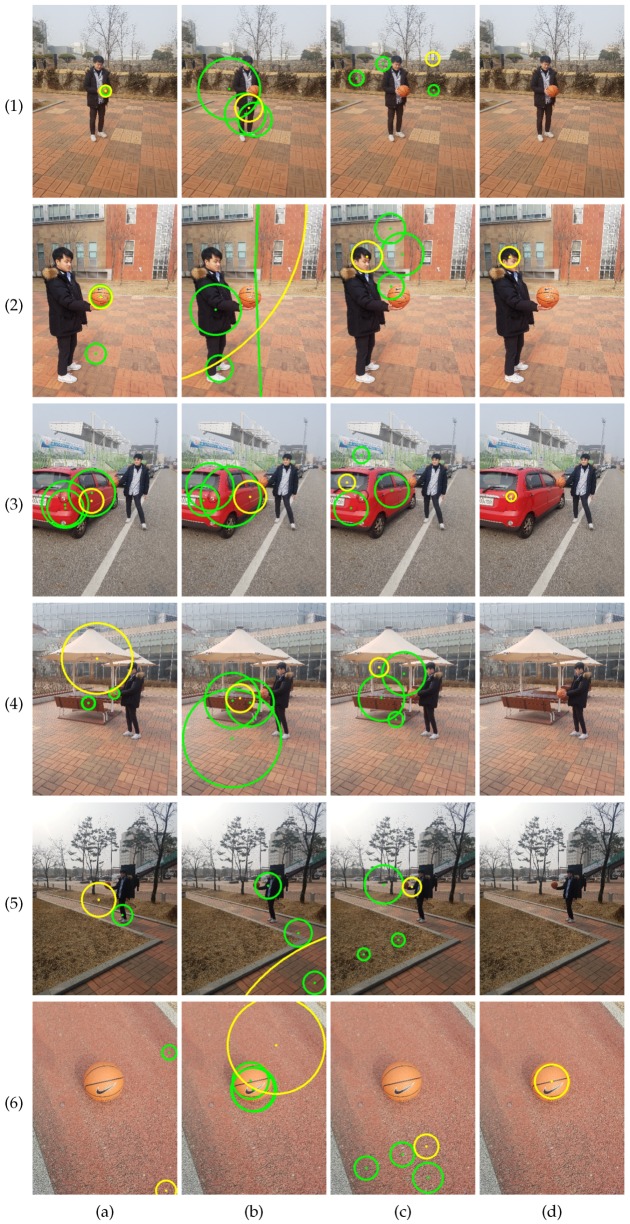
Top four circles detected by the methods (basketball set). (**a**) Proposed method. (**b**) Kwon et al. [21]. (**c**) CHT [36]. (**d**) EDCircles [44]. From (**a**–**d**), the best circles are drawn in yellow, while the remaining circles are drawn in green. Some circles have similar centers and radii in (**a**,**b**), so the numbers of circles in the images may seem less than four. Best viewed in color.

Figure 9 and Figure 10 show sample results on the gym ball set and the basketball set, respectively. Figure 9(1), (2), and (3) show the failure cases of EDCircles, while Figure 9(4), (5), and (6) show failure cases of the proposed method. Due to the lack of color information, EDCircles detected human heads or shadows of the ball as a circle. In contrast, the proposed method mistook red circular regions as the calibration object. The results in Figure 10 show a similar tendency to those in Figure 9. When the sphere was too small, all four methods failed, as shown in Figure 10(5).

The use of color information helps prevent general circular shapes from being detected as the specially-colored calibration object. However, relying too much on the color information tends to be the source of performance degradation under illumination changes. The proposed method relies on both color and edge information, which enables balancing robustness and false detection. We note that we checked the results of EDCircles with its false rejection disabled. There was no noticeable difference from the results reported in this paper.

### 4.2. Extrinsic Calibration Results

**Figure 11:**
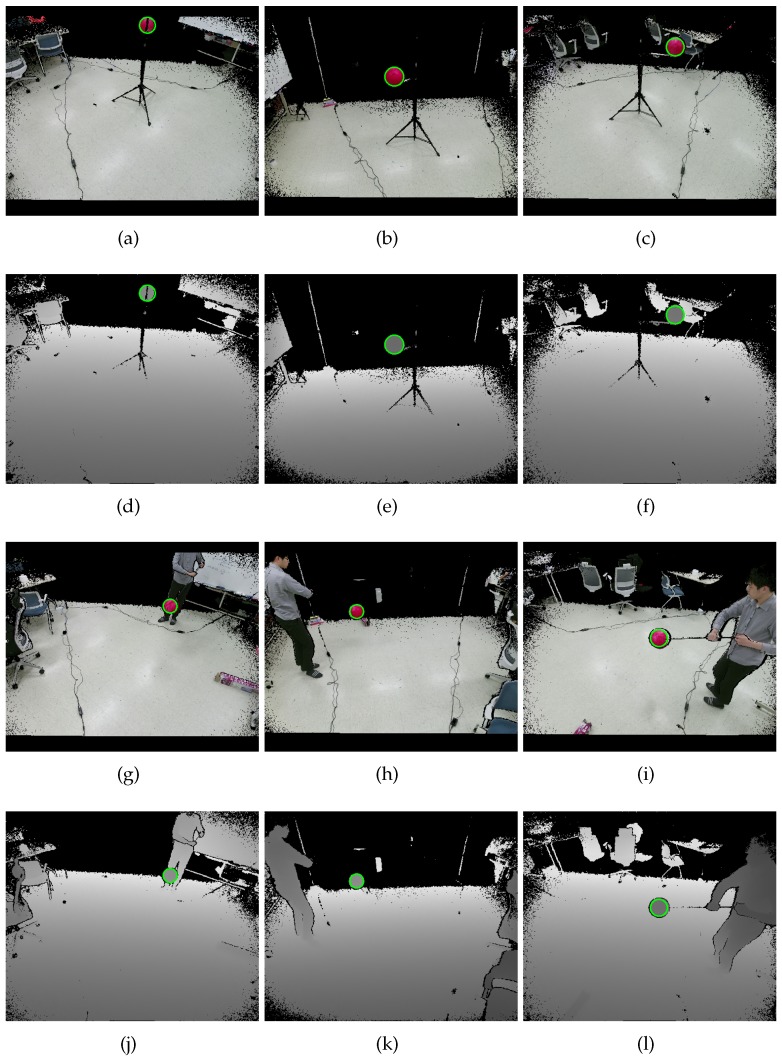
Examples of aligned and undistorted color and depth image pairs acquired by three Kinect v2 cameras. From (**a**–**f**), we have collected a static set by capturing still images of a static sphere. From (**g**–**l**), we have collected a dynamic set by capturing videos of a sphere carried by a person. The green empty circles represent sphere regions detected by the proposed method.

The proposed method is applicable to any factory-calibrated RGB-D cameras as long as the color camera has negligible radial distortion. In this section, we provide experimental results on datasets acquired by Kinect v2 cameras [6]. We have chosen to use Kinect v2 cameras [6] for the following reasons. The size of our lab is 3.6 m × 3.9 m, and Kinect v2 cameras’ reliable working range is 4.5 m. In addition, Kinect v2 cameras provide aligned pairs of color and depth images, as shown in Figure 11. The depth cameras equipped in RGB-D cameras usually have large radial distortion; however, the aligned image pairs provided by Kinect v2 are undistorted ones. The accuracy of the intrinsic and extrinsic parameters of individual RGB-D cameras is important for accurate 3D reconstruction. However, the analysis of the accuracy of the factory calibration is out of the scope of this paper.

**Figure 12:**
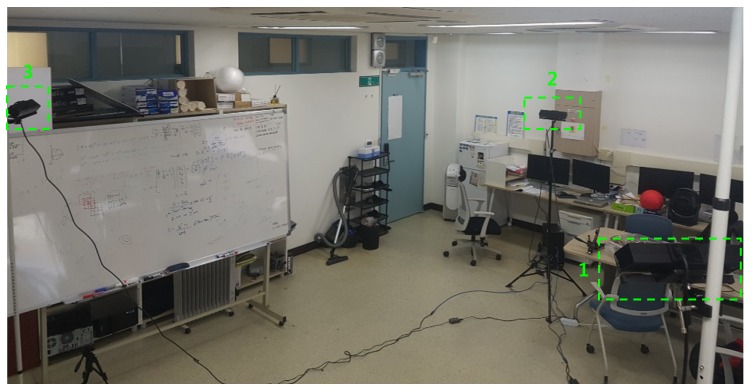
Our Kinect v2 camera setup.

**Figure 13:**
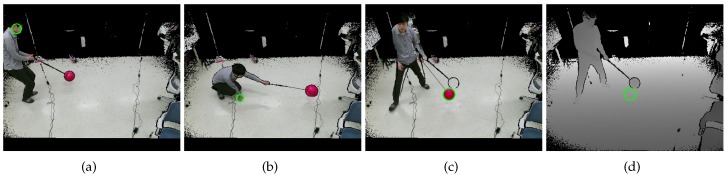
(**a**,**b**) show examples of misdetected circles. In (**c**), the circle has been accurately detected. However, in (**d**), the circular region does not match the sphere region in the corresponding depth image due to the asynchronous nature of the Kinect v2 camera.

As shown in Figure 12, our capturing system consists of three sparsely-placed Kinect v2 cameras surrounding the middle of the room. All three cameras were connected to a single computer with an Intel Core i7-4790 processor and 8 GB of RAM, running Windows 10. We let three threads capture three pairs of color and depth images simultaneously; however, perfect synchronization could not be achieved due to the asynchronous nature of Kinect v2 cameras, as shown in Figure 13c,d.

For the experiments, we acquired three sets of color and depth image pairs. The first two sets consist of images of a red styrofoam ball, whose radius is 12.5 cm. Because a large sphere provides more surface points for sphere fitting, a large sphere is preferred. Our styrofoam ball was the largest and lightest among commercially-available ones. We mainly used the first two sets for the analysis of the proposed method.

The first set is a static set with perfect synchronization. To collect such perfectly-synchronized color and depth image pairs, we used a stand to fix the sphere, as shown in Figure 11a–f. Still images of the stand were taken by manually pressing a button. The number of color and depth image pairs was 95 (N=95) per camera, and the proposed method missed only one sphere center in the entire set. Since the visibility of the missing sphere was automatically set to zero, the static set included no outliers.

The second set was a dynamic set collected by capturing videos of the styrofoam ball carried by a person. The set consisted of 164 color and depth image pairs per camera (N=164), with tens of missing spheres and misdetected spheres. Figure 13 shows examples of misdetected spheres. In the $a^{*}b^{*}$ color space, the skin color is similar to the color of our sphere. In addition, the face and hand seem circular from a distance. Therefore, the proposed method sometimes mistook the face and hand as the sphere, as shown in Figure 13a,b. Another source of error is the asynchronous nature of the Kinect v2 camera, as shown in Figure 13c,d.

The third set (static basketball set) consists of still images of a basketball. This set was acquired to show the robustness of the proposed method to changes in calibration objects. We have already shown that the basketball can be detected by the proposed method without parameter tuning. In this subsection, we show that it can be actually used for the extrinsic calibration. The set consists of 101 color and depth image pairs per camera (N=101).

For comparison, we implemented the extrinsic calibration method by Su et al. [20]; for brevity, the implementation is denoted as Su et al. or Su et al.’s method. Because our datasets do not include background images, we used the same sphere centers as ours. To implement the pose refinement part of their alternating optimization, we used the levmar library [46]. The bundle adjustment of our proposed method also relies on the same library.

**Figure 14:**
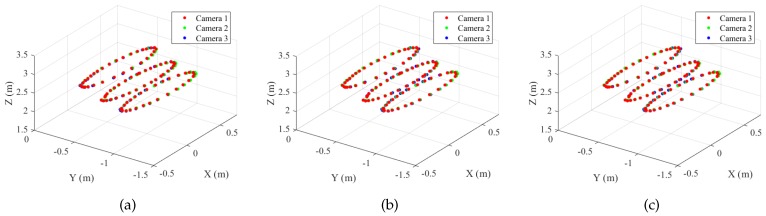
Aligned sphere centers (static set, N=95). (**a**) Proposed method (pairwise). (**b**) Proposed method (Bundle Adjustment (BA)). (**c**) Su et al. [20]. Refer to the text for more detail. Best viewed in color.

Figure 14 shows the trajectories of the sphere centers of the static set. For each method, all the sphere centers in camera *q* have been transformed to the reference frame. As shown in the figure, the pairwise pose estimation (pairwise) and bundle adjustment (BA) of the proposed method registered the trajectories from different cameras accurately. Su et al.’s method also showed the same alignment as BA.

**Figure 15:**
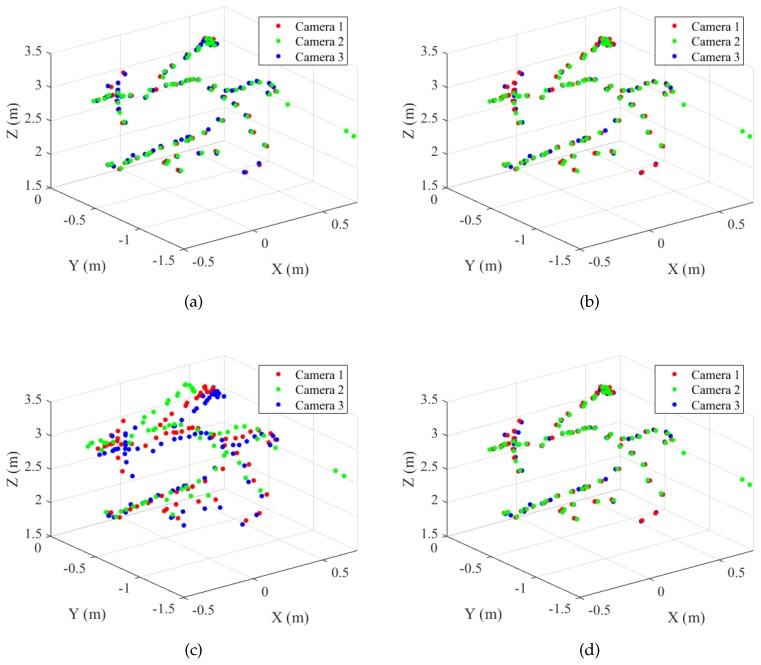
Aligned sphere centers (dynamic set, N=164). (**a**) Proposed method (pairwise). (**b**) Proposed method (BA). (**c**) Su et al. [20]. (**d**) Su et al. [20] (inlier set). Refer to the text for more detail. Best viewed in color.

Figure 15 shows the trajectories of the sphere centers of the dynamic set. The proposed method shows good alignment between the trajectories; however, Su et al.’s method failed to estimate accurate transformations because of the inaccurate sphere centers (some of them shown in Figure 13). To remove the outliers, we extracted the inliers to the camera poses estimated by the proposed method (BA) with an error threshold of 12 cm and applied Su et al.’s method to the inlier set. The method is denoted as Su et al. (inlier set), which gives equivalent results as the proposed method, as shown in Figure 15d.

**Figure 16:**
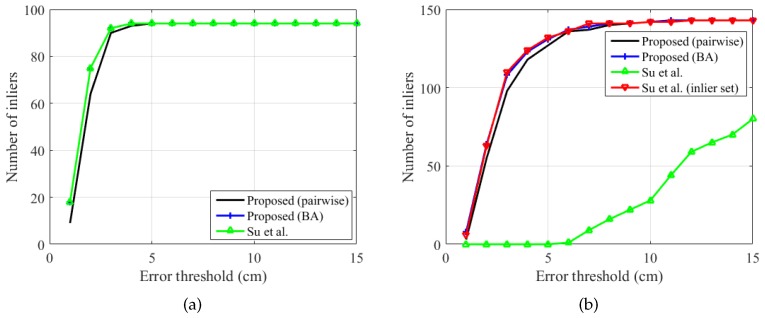
Number of inliers. The figures show the number of triples of corresponding sphere centers whose maximum pairwise distance is less than the error threshold. (**a**) Static set (N=95). (**b**) Dynamic set (N=164). Refer to the text for more detail. Best viewed in color.

Figure 16a shows the number of inliers according to the error threshold. Here, the number of inliers is defined as the number of triples of corresponding sphere centers whose maximum pairwise distance is less than the error threshold:

(15) $$\mathrm{Number}\mathrm{of}\mathrm{inliers}=\sum_{i=1}^{N}1(\max\left(∥C_{i}^{\left(1\right)}-{\tilde{C}}_{i}^{\left(2\right)}∥,∥C_{i}^{\left(1\right)}-{\tilde{C}}_{i}^{\left(3\right)}∥,∥{\tilde{C}}_{i}^{\left(2\right)}-{\tilde{C}}_{i}^{\left(3\right)}∥\right)<\mathrm{Error}\mathrm{threshold}),$$

where the first camera is the reference camera and 1(x) is a function returning one if *x* is true and otherwise zero. ${\tilde{C}}_{i}^{\left(2\right)}$ and ${\tilde{C}}_{i}^{\left(3\right)}$ are the sphere center positions transformed from the second and the third camera to the reference frame. All the maximum distances were less than 4 cm for the proposed method (BA) and Su et al.’s method. In addition, 98% of the maximum distances were less than 3 cm for the two methods.

Figure 16b shows the number of inliers of the dynamic set. The proposed method (BA) and Su et al. (inlier set) showed similar results, while the original Su et al.’s method showed only a small number of inliers, even with large error thresholds.

**Figure 17:**
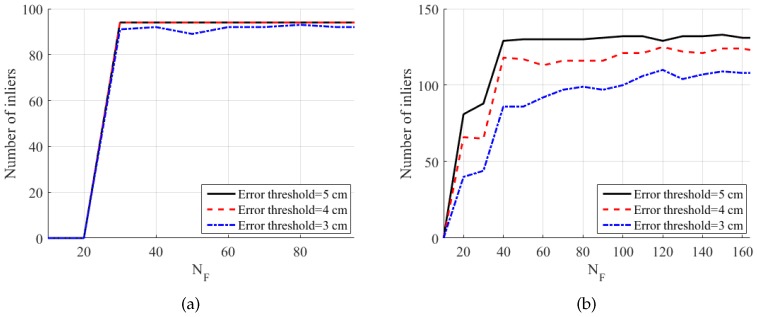
Number of inliers according to the number of sphere centers $N_{F}$ used for extrinsic calibration (proposed method). The figures show the number of triples of corresponding sphere centers whose maximum pairwise distance is less than the error threshold. (**a**) Static set (N=95). (**b**) Dynamic set (N=164). Refer to the text for more detail. Best viewed in color.

To analyze the effect of the number of points used for extrinsic calibration, we conducted our proposed extrinsic calibration procedure using only the first $N_{F}$ frames and tested the accuracy of the calibration, as shown in Figure 17. Figure 17a shows the result on the static set. The sphere in the first 29 frames of the static set was rotated on a plane, which is the reason for the abrupt increase in the number of inliers at $N_{F}=30$. When we acquire still images of the spherical object, it is more important to avoid degenerate configurations than just to increase the number of images. Given a single sphere center outside the plane, the number of inliers immediately converges to the maximum value. In contrast, when we acquire videos of a freely-moving sphere, a large number of frames is helpful, as shown in Figure 17b. The number of inliers, with an error threshold of 5 cm, converges at $N_{F}=40$; however, with an error threshold of 3 cm, it reaches the maximum at $N_{F}=120$. We conjecture that the difference between the static set and the dynamic set is caused by the imperfect synchronization of our system.

**Figure 18:**
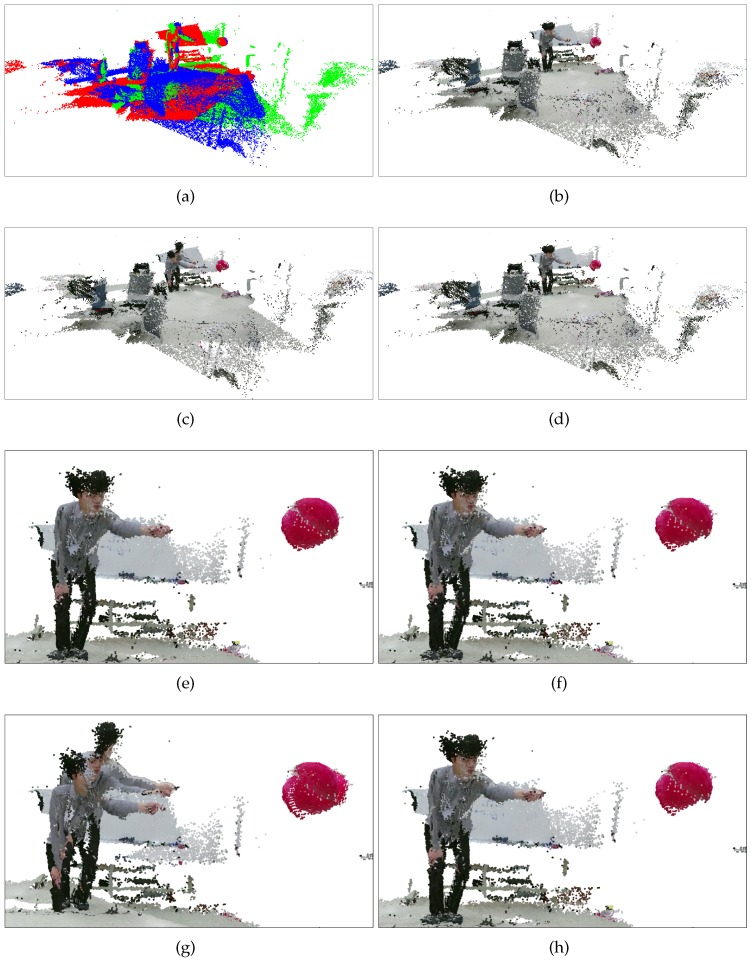
Rendering results (dynamic set). (**a**) Overview of the environment. 3D point clouds from different cameras have been drawn in different colors. (**b**) Proposed method (BA). (**c**) Su et al. [20]. (**d**) Su et al. (inlier set). (**e**) Proposed method (pairwise). (**f**) Proposed method (BA). (**g**) Su et al. [20]. (**h**) Su et al. [20] (inlier set). Refer to the text for more detail. Best viewed in color.

Figure 18 shows the fused 3D point clouds attained by the methods. The depth images fused together are from the dynamic set. The proposed methods (pairwise and BA) and Su et al. (inlier set) show visually similar results, while Su et al. fails in aligning the point clouds, as shown in Figure 18c,g.

**Figure 19:**
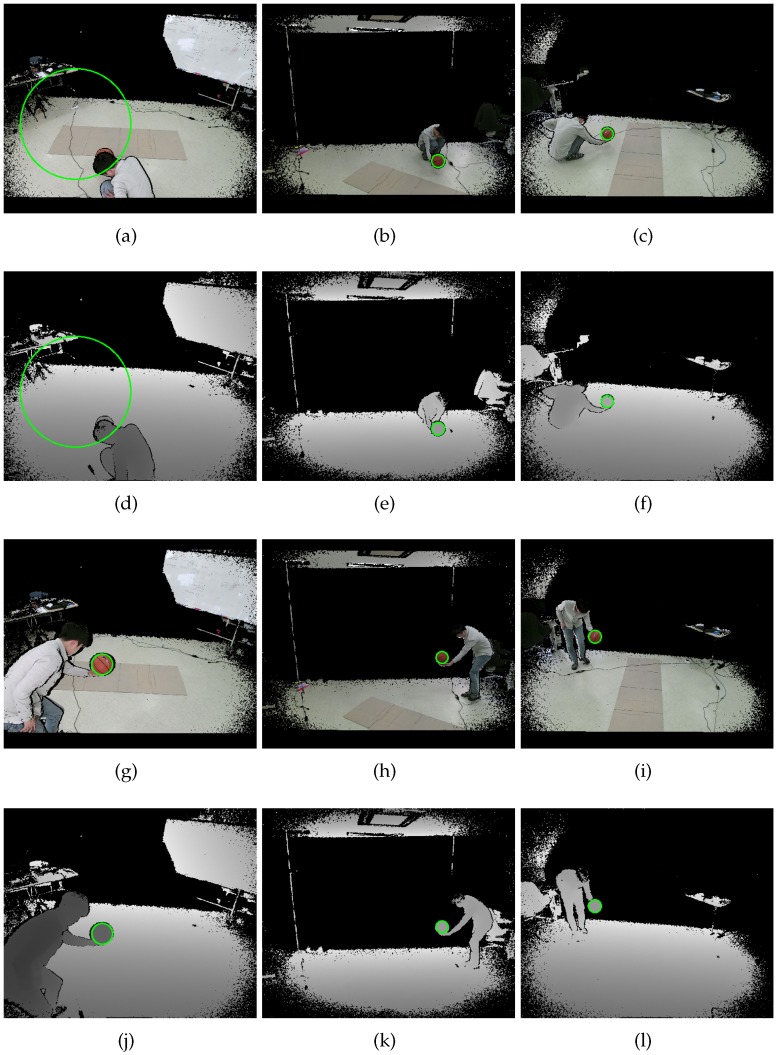
Examples of aligned and undistorted color and depth image pairs of the static basketball set. The green empty circles represent sphere regions detected by the proposed method. (**a**), (**b**), (**c**) show corresponding color images, and (**d**), (**e**), (**f**) show their corresponding depth images. (**g**), (**h**), (**i**) show another triple of corresponding color images, and (**j**), (**k**), (**l**) show their corresponding depth images.

Figure 19 shows color and depth image pairs from the static basketball set. The circle detection results showed a similar tendency to the static and dynamic sets. In Figure 19a, the proposed circle detection method finds a nonexistent circle due to occlusion. The proposed method can use Equation (7) to reject such nonexistent circles. We, however, relied on robust cost functions in the extrinsic calibration procedure because the rejection requires another threshold and never will be perfect. If the calibration object does not exist in the majority of the images, such a rejection method would be necessary.

**Figure 20:**
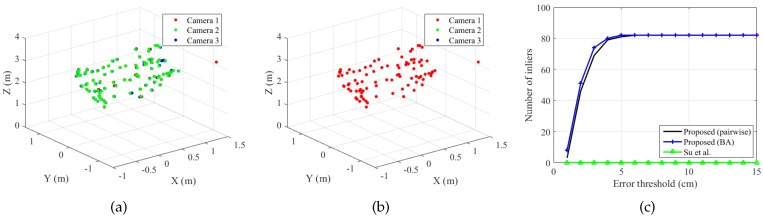
Aligned sphere centers and number of inliers (static basketball set, N=101). (**a**) Proposed method (BA). (**b**) Su et al. [20]. (**c**) Number of inliers. Refer to the text for more detail. Best viewed in color.

**Figure 21:**
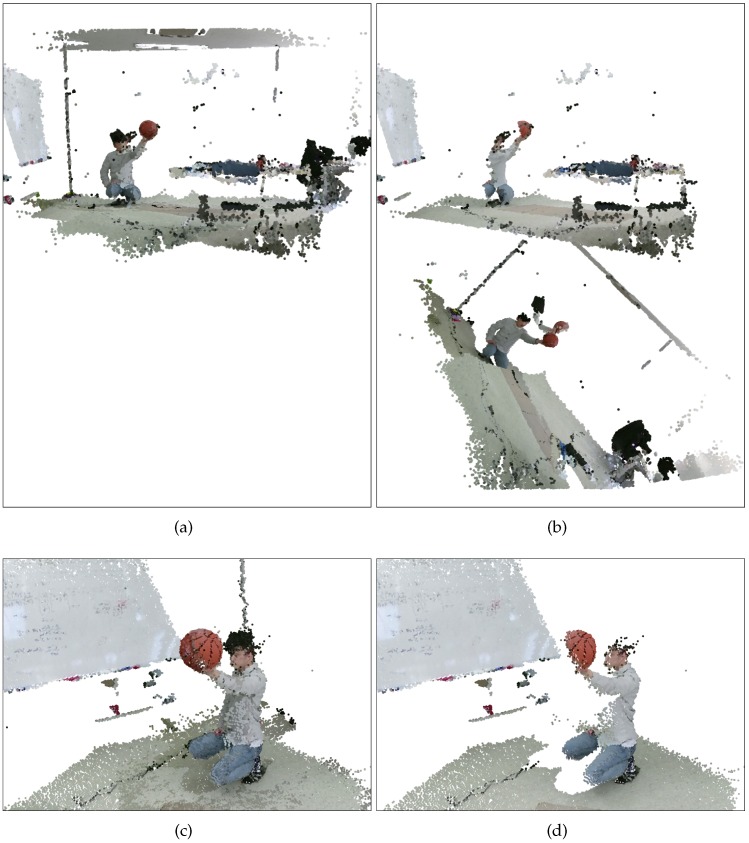
Rendering results (static basketball set). (**a**,**c**) Proposed method (BA). (**b**,**d**) Su et al. [20]. Refer to the text for more detail. Best viewed in color.

Figure 20 shows the calibration accuracies on the basketball set. The curve shapes of the proposed method in Figure 20c are very similar to the static set. We conjecture that this is because we minimized the synchronization problem by acquiring still images of the basketball. In contrast, the curve shape of Su et al. [20] was completely different due to the presence of outliers. Figure 20b shows that the sphere centers in Cameras 2 and 3 are not accurately transformed to the reference frame. The calibration accuracies can be also compared in Figure 21, which shows fused 3D point clouds attained by the methods.

### 4.3. Computation Time

We measured the computation of the proposed method and Su et al.’s method, using a computer with an Intel Core i5-7300U processor and 4 GB of RAM, running Windows 10. We ran the two methods 100 times, and Table 2 shows the average computation time. The computation time of the proposed pairwise pose estimation was about 10,000 times longer than that of Su et al. This is because the proposed method relied on 10,000 random samples to determine the best pose between cameras. The alternating optimization algorithm for the bundle adjustment by Su et al. is highly efficient and about 10-times faster than the proposed method. This is the main drawback of the proposed method.

## 5. Conclusions

In this paper, we proposed a fully-automated method for extrinsic calibration of multiple RGB-D cameras. The proposed method uses a monochromatic spherical object as the calibration target, and we proposed a method for detecting the object based on the assumption that its projected region is circular. Robustness to background changes or lighting changes is an important quality of the detection method. Excessive robustness, however, can lead to false detection of the calibration object. Our circle detection method was designed to balance the robustness and the false detection, so it is possible to use a similar object such as a basketball in place of the learned calibration object, without parameter tuning.

On the other hand, outliers are inevitable in any practical pattern recognition system. The proposed circle detection method is ready to reduce the outliers at various stages; however, the parameters for the rejection may fail in other objects or environments. Our choice was to rely on robust loss functions so that a point set with outliers can be safely used for the extrinsic calibration. The experiments have shown that the proposed method is robust to misdetected sphere centers. The experiments also have shown that it is important to avoid degenerate configurations and that it is important to increase the number of frames to overcome the imperfect synchronization.

A drawback of the proposed method is that its bundle adjustment is inefficient. Using RGB-D cameras connected to their own computers, it is possible to deploy the sphere detection and pairwise pose estimation steps to the computers so that the main computer will conduct only the bundle adjustment. In this case, the bundle adjustment is indeed the bottleneck. More research will be done in our future work to reduce computational complexity.

## Figures and Tables

**Table 1:** Summary of the parameters used in this paper. The mean sphere color has been computed from OpenCV $CIE-La^{*}b^{*}$ images. $a^{*}$ and $b^{*}$ range from 0–255. MSAC, M-estimator Sample Consensus.

Parameter	Stage or Meaning	Setting in This Paper	Recommended Settings
τ (or $\tau^{2}$)	Error-clipping value (threshold) of the robust loss function ρ		
$\tau_{CF}$	Circle fitting	3 pixels	2–4 pixels
$\tau_{MC}$	Circle detection	$\min(\tau_{CF},\;0.1$ × circleradius)	Adaptive
$\tau_{SF}$	Sphere fitting	2 cm	1–5 cm
$\tau_{RT}$	Pairwise pose estimation	$2\sqrt{3}$ cm	$\sqrt{3}\tau_{BA}$
$\tau_{BA}$	Bundle adjustment	2 cm	1–5 cm
$N_{S}$	Number of total samples in MSAC		
$N_{S}$	Circle fitting	1000	1000
$N_{S}$	Sphere fitting	10,000	10,000
$N_{S}$	Pairwise pose estimation	10,000	10,000
$a_{m}=\left(a_{m}^{*},b_{m}^{*}\right)$	Mean sphere color	(165.79, 146.02)	Learned
*K*	Hierarchical segmentation	30	30
$d_{\min}$	Circle detection	10	5–15 (a small value)
$P_{\min}$	Circle detection	10%	Dependent on the purpose
$r_{\min}$	Circle detection	10 pixels	10 pixels
$r_{\max}$	Circle detection	0.5·min(imagewidth,imageheight)	Adaptive

**Table 2:** Average computation time of the proposed method and Su et al. [20].

Method	Stage	Static Set (N=95)	Dynamic Set (N=164)
Proposed	Circle detection (per image)	53.5 ms	60.5 ms
Proposed	Sphere fitting (per region)	327 ms	313 ms
Proposed	Pairwise pose estimation (per camera pair)	776 ms	1.28 s
Proposed	Bundle adjustment	29.6 s	149 s
Su et al. [20]	Pairwise pose estimation (per camera pair)	78.3 μs	82.3 μs
Su et al. [20]	Bundle adjustment	2.09 s	3.51 s
